# Endometriosis and Sexual Functioning: How Much Do Cognitive and Psycho-Emotional Factors Matter?

**DOI:** 10.3390/ijerph19095319

**Published:** 2022-04-27

**Authors:** Valentina Rossi, Roberta Galizia, Francesca Tripodi, Chiara Simonelli, Maria Grazia Porpora, Filippo Maria Nimbi

**Affiliations:** 1 Institute of Clinical Sexology, 00198 Rome, Italy; francitrip@hotmail.com; 2 Department of Dynamic, Clinical and Health Psychology, “Sapienza” University, 00185 Rome, Italy; roberta.galizia@uniroma1.it (R.G.); chiara.simonelli@uniroma1.it (C.S.); filippo.nimbi@uniroma1.it (F.M.N.); 3 Department of Maternal and Child Health and Urology, “Sapienza” University, 00161 Rome, Italy; mariagrazia.porpora@uniroma1.it

**Keywords:** endometriosis, dyspareunia, sexual dysfunctions, chronic pelvic pain, genital pain, emotions, cognitions

## Abstract

Women with endometriosis often suffer from genito-pelvic pain. The objective of the present study was to analyze the relationship between cognitive and psycho-emotional factors and sexual functioning of women with endometriosis, comparing women with and without endometriosis. A total of 87 women with endometriosis (EG) and 100 women without endometriosis (CG) completed a socio-demographic questionnaire; the short-form of McGill Pain Questionnaire (SF-MPQ); the Female Sexual Functioning Index (FSFI); the Sexual Distress Scale (SDS); the Symptoms Checklist (SCL-90-R); the Toronto Alexithymia Scale (TAS-20); the Positive and Negative Affects Scale (PANAS); the Sexual Dysfunctional Belief Questionnaire (SDBQ); the Sexual Modes Questionnaire (SMQ); and the Questionnaire of Cognitive Schema Activation in Sexual Context (QCSASC). EG obtained worse scores than CG in SF-MPQ, Pain subscale of FSFI, and SDS. EG reached higher scores than CG on almost all scales of SCL-90-R and lower scores in the “Identifying Feelings” scale of TAS-20. Furthermore, EG reported more negative emotions toward sexuality than CG (PANAS) higher scores on the “Affection Primacy” scale of SDBQ and the “Helpless” sexual cognitive schema of QCSASC than CG. No significant differences were found in SMQ. Sexual health professionals should address psychological, emotional, and cognitive factors since they seem involved in patients’ sexual experiences.

## 1. Introduction

Endometriosis is a chronic gynecological disease characterized by the presence and the growth of endometrial-like tissue outside the uterine cavity [1]. Approximately 7–10% of reproductive-aged women appear to be affected by endometriosis, although the exact prevalence rate remains unknown [2,3]. Chronic pelvic pain (CPP)—which includes dysmenorrhea, dyspareunia, dyschezia, and dysuria—is the most common symptom in women with endometriosis and occurs up to 80% of the cases [4]. However, symptomatology can differ from woman to woman: about 20–25% of patients are asymptomatic, and about 30–50% suffer from infertility [2,5,6]. Endometriosis lesions are anatomically classified as superficial peritoneal endometriosis (SUP); ovarian endometriomas (OMA); deep infiltrating endometriosis (DIE) [7]. Etiopathogenesis is still controversial [8], and no definitive cure is available [9].

Because of its symptomatology, risk of infertility, delayed diagnosis, lack of definitive treatment, recurrence of the condition, and impact on female identity, endometriosis has a profound impact on quality of life (QoL) [10,11,12,13,14,15,16,17,18,19]. It affects patients’ daily activities, education, employment, relationships, and sexuality [11,13,14,15,16,17,19,20,21,22,23,24,25,26,27]. Mental health may be severely compromised [11,28,29]: anxiety and depression are the most common problems reported by women [14,17,19,28,29,30,31,32]. Somatization, alexithymia, interpersonal sensitivity, obsessive compulsive, and phobic anxiety are also described [28,29,30,33,34].

Regarding sexuality, dyspareunia is the most common complaint [11,13,21,23,35], affecting 32–70% of patients [36]; however, difficulties in all phases of sexual response are widely reported in the literature [10,11,18,21,23,35,37,38,39,40,41,42]. The prevalence of sexual problems increases when sexual distress is considered; which is observed in women with endometriosis more than sexual symptoms [11,41,43].

The pain appears to play an essential role in patients’ sexual health [10,11,13,14,15,16,17,19,21,22,36,44,45,46,47,48,49]. In this regard, the biopsychosocial (BPS) model suggests the consideration of the different aspects—biological, psychological, and socio-relational—that, interacting with each other, could influence a patient’s health [12,21,23,34,36,39,40,41,44,48,50,51,52,53,54]. Moreover, pain is not only characterized by a somatic component but also involves cognitive and emotional components. A “vicious circle” in the experience of pain, with the mutual involvement and interaction of biopsychosocial factors, has been assumed in recent years [29,30,32,55,56].

In light of this consideration, interest in the association between cognitive and emotional factors, sexual dysfunctions and sexual distress, and pain perception in women with endometriosis has grown exponentially. Negative beliefs predict sexual distress beyond pain symptoms [43]. Specifically, women with negative cognitions might cope negatively with the stressor (e.g., pain and sexual problems) and might be more sexually distressed than those without dysfunctional beliefs. In addition, negative cognitions (e.g., pain catastrophizing) [57,58,59,60], specific coping styles (e.g., passive) [59,60], personality or temperament traits (e.g., worry traits, deep pessimistic concerns, external locus of control, negative beliefs about pain control) [60,61,62], presence of anxious and depressive symptoms [32,57,60], and suppression of emotions [59] could negatively affect the experience of pain.

Although the literature about the influence of cognitive and emotional factors on sexual function and pain perception has increased in recent years, the presence of some aspects, such as cognitive sexual schemas, beliefs, automatic thoughts, and emotions toward sexuality, remain unexplored in women with endometriosis. Nevertheless, it has been shown that these factors play an important role in the development and maintenance of all sexual dysfunctions [63,64,65,66,67,68,69,70,71,72]. A cognitive-emotional model has been described only for vaginismus, sexual desire and orgasm disorders, and sexual dissatisfaction [73,74,75,76]. There are no available data on women with endometriosis-related genito-pelvic pain (GPP) to the best of our knowledge.

The general purpose of the study was to investigate if cognitive and psycho-emotional factors influence the sexual functioning of patients with endometriosis. This general aim was composed of three specific objectives. The first objective was to compare the sexual function of women with and without endometriosis. The hypothesis was that women with endometriosis were more sexually dysfunctional and distressed than women without the pathology. The second aim was to assess the psycho-emotional profile of these women, analyzing the presence of psychopathological traits and symptoms, the difficulty in recognizing and expressing emotions, and the presence of negative emotions related to sexuality. It was assumed that women with endometriosis had a worse psycho-emotional profile than women without it. Lastly, the study explored differences in cognitive schemas, automatic thoughts, and beliefs related to sexuality with the hypothesis that women with endometriosis show more negative cognitions than women without this condition.

## 2. Materials and Methods

The study was conducted on 187 women: 87 patients with endometriosis (Endometriosis Group—EG), recruited in the [omitted for blinded review] (34.10 ± 9.18; ranged 18–56 years), and 100 from the general population (Control Group—CG) (34.58 ± 9.27; ranged 18–61 years), recruited using snowball methodology. Both groups completed an anonymous web survey on the Google. Docs platform assessing psycho-social domains related to female sexuality. The questionnaires were all self-administrated for both groups.

For the CG, the web survey was disseminated on institutional websites and social networks (e.g., Facebook, Instagram, and LinkedIn). In turn, participants were asked to share the study on their own social network or with women interested to participate. The inclusion criteria were self-declaring as a woman, identifying as heterosexual, and being a fluent Italian speaker. Later, only women matching with those of the EG were selected for the study. Completion of the questionnaire took approximately 25 min. No remuneration was provided to participants.

A multidisciplinary team of gynecologists and psycho-sexologists was involved for EG. Gynecologists recruited eligible participants during consultations, and the psycho-sexologists explained the study’s objectives and gave the link for the Google. Docs platform. Patients completed the questionnaire alone, accessing it at home. Inclusion criteria for the EG were: having undergone laparoscopic treatment with histologic confirmation at least three months before the study; taking oral contraceptives or progestogens. The diagnosis of endometriosis was confirmed for all participants by gynecological examination and transvaginal pelvic ultrasound. Exclusion criteria were: not identifying as heterosexual, DIE presence, and treatment with gonadotropin releasing hormone (GnRH) analogues.

All subjects gave their informed consent for inclusion before participating in the study. The study was conducted following the Declaration of Helsinki, and the protocol was approved by the Ethics Committee of [omitted for blind review] on 20 September 2017. Data were collected from November 2017 to September 2019.

In line with the aim of the present study, 9 self-report measures were administered based on their validity, clarity, and extensive use in the literature. The selected measures are listed here below.

An ad hoc sociodemographic form to collect general information, such as age, relationship, and marital status, educational level, work status, children, self-declaration of having any sexual problem (i.e., “During the past 6 months, have you had any sexual problem?” and “How long have you had this sexual problem?), help-seeking behavior, and treatments.

The Short Form of McGill Pain Questionnaire (SF-MPQ) [77] is composed of 15 items (11 sensory and 4 affective) rated on an intensity scale from “none” to “severe.” This questionnaire collects both quantitative and qualitative information related to the subjective experience of pain. A total score can be calculated as an indicator of pain intensity. For this study, the SF-MPQ adaptation to GPP by Nimbi and colleagues [12] was used. The SF-MPQ was assessed only in women answering “yes” to the question “In the last 6 months, have you experienced any kind of pain/discomfort located in the genital area (in general or associated with sexual activity)?” The Cronbach’s alpha value for the adapted measure in the present study was 0.87.

The Female Sexual Functioning Index (FSFI) [78,79] is a 19-item questionnaire providing information on the general sexual functioning and, in particular, on six specific dimensions: desire, arousal, lubrication, orgasm, sexual satisfaction, and sexual pain. Higher scores indicate higher sexual functioning. The measure presents acceptable test–retest reliability, internal consistency, validity, and the ability to discriminate between sexually clinical and healthy women [79]. The total score ≤ 26.55 indicates the presence of sexual dysfunctions [79,80]. In this study, Cronbach’s alpha values for this measure ranged from 0.81 (arousal) to 0.93 (pain).

The Sexual Distress Scale (SDS) [81] is a 12-item questionnaire assessing personal distress related to sexuality. Items converge into a single factor structure (total distress). Test–retest reliability and internal consistency coefficients are acceptable. The measure can discriminate between sexually functional and dysfunctional subjects [81]. The Cronbach’s alpha value for this measure in the present study was 0.91.

The Symptom Check List-90-Revised (SCL-90-R) [82,83] is a widely used self-report measure assessing the severity of psychopathological symptoms on a 5-point Likert scale ranging from “not at all” to “extremely.” The SCL-90-R consists of three global indexes (Global Severity Index, Positive Symptom Total, and Positive Symptom Distress Index) and nine symptomatologic subscales exploring the condition during the previous 7 days: Somatization, Obsessive Compulsive (OC), Interpersonal Sensitivity (IS), Depression, Anxiety, Hostility, Phobic Anxiety, Paranoid Ideation, and Psychoticism. The validity of the SCL-90-R has demonstrated good internal consistency [83]. The Cronbach’s alpha values for this measure in the present study ranged from 0.85 (psychoticism) to 0.92 (somatization).

The Toronto Alexithymia Scale-20 (TAS-20) [84,85] is a 20-item measure assessing a general dimension of alexithymia and three main factors (Difficulty Identifying Feelings, Difficulty Describing Feelings, and Externally Oriented Thinking). The TAS-20 has demonstrated adequate internal and test–retest reliability [85]. The Cronbach’s alpha values for this measure in the current study ranged from 0.78 (difficulty describing feelings) to 0.87 (externally oriented thinking).

The Positive and Negative Affect Schedule (PANAS) [86,87] is a 20-item self-report measure of positive and negative emotions. Participants are asked to rate their experience related to each emotion (such as being determined, active, interested, attentive, enthusiastic, focused, strong, inspired, excited, proud, frightened, upset, nervous, frightened, distressed, guilty, ashamed, irritable, and hostile) at a specific time or situation (in this study, during sexual activity) on a 5-point scale (1—very little or not at all to 5—very much). Higher scores indicate greater emotional compliance. Psychometric studies have reported good reliability, validity, and ability to discriminate between depression and anxiety in clinical samples [87]. The Cronbach’s alpha values for this measure in the present study ranged from 0.84 (positive affects) to 0.86 (negative affects).

The Sexual Dysfunctional Belief Questionnaire (SDBQ) [88,89] is a 40-item questionnaire that assesses classes of beliefs about female sexuality: Sexual Conservatorism, Affection Primacy, Control Over Sexuality, Age-related Beliefs. Responses are rated on a 5-point Likert scale (from 1 = “completely disagree” to 5 = “completely agree”). Higher scores indicate more dysfunctional sexual beliefs. Good coefficients of test–retest reliability, internal consistency, and discriminant validity were shown [89]. Cronbach’s alpha values in the current study ranged from 0.78 (control over sexuality) to 0.87 (sexual conservatorism).

The Sexual Modes Questionnaire [90,91] is a 30-item questionnaire to measure the connection between automatic thoughts, emotions, and sexual responses in a sexual context. The Italian female’s version consists of 6 factors: sexual abuse thoughts, lack of erotic thoughts, low self-body image thoughts, failure and disengagement thoughts, sexual passivity and control, and partner’s lack of affection. Higher scores in each dimension are related to the presence of more negative thoughts. Psychometric studies supported test–retest reliability and internal consistency, and discriminant validity [91]. Cronbach alpha values in the current study ranged from 0.79 (partner’s lack of affection) to 0.9 (failure and disengagement thoughts).

The Questionnaire of Cognitive Schema Activation in Sexual Context (QCSASC) [92,93] is a 28-item questionnaire that measures cognitive self-schemas activated in problematic sexual situations. Concerning the most recurrent sexual distressful episode, the respondent is asked to rate on a 5-point Likert scale (1—“completely false” to 5—“completely true”), the degree of agreement with 28 thoughts. In the Italian version [93], two domains were identified: “Helpless” and “Unlovable” self-schemas. The QCSASC showed good internal consistency, test–retest reliability, convergent validity, and incremental validity [93]. Cronbach alpha values in the current study ranged from 0.89 (helpless) to 0.91 (unlovable).

Statistical significance for all tests was determined a *p* < 0.05.

Data were analyzed conducting descriptive and multivariate analyses with the Statistical Package for Social Sciences v22.0 (SPSS, Chicago, IL, USA). Chi-square test was used to evaluate differences among sociodemographic variables. To test significant differences between the two groups and the sexual, psychological, emotional, and cognitive variables ANOVAs and MANOVAs were run.

## 3. Results

The sociodemographic characteristics of the participants divided into EG and CG are presented in Table 1. The mean age of the participants of the CG was 34.58 ± 9.27 (range 18–61), and the mean age of the EG was 34.10 ± 9.18 (range 18–56). Groups differed significantly in three variables: “Relationship Status”, “Desire to Have a Baby”, and “Sexual Problems in the last 6 months”. Both groups were predominantly in a relationship; however, EG women were more often in a relationship than CG. Moreover, EG mostly declared to desire to have a baby at the moment of the study; instead, the majority of CG women declared not to have this desire at the moment of the study. Moreover, EG showed higher rates of sexual difficulties in the last 6 months compared to CG. Only 24.1% of EG who reported sexual problems referred to sexual health professionals or were undergoing therapy for those complaints at the moment of the study.

To explore the differences between the two groups in some sexual, psychological, emotional, and cognitive domains, one-way ANOVAs and MANOVAs were run separately for each domain. Results are shown in Table 2.

About sixty-nine percent of EG women (68.96%) reported suffering from genital pain (GP) compared to 6% of CG. Regarding the intensity of the pain (SF-MPQ) among the women who reported GP, the two groups presented significant differences: EG reached higher scores than CG, meaning a higher intensity of the painful symptomatology.

Considering sexual function measured with the FSFI, EG, and CG were not significantly different. Both groups presented scores under the cut-off point (≤26.55), showing the presence of sexual dysfunctions. The two groups showed significant differences only in the Pain subscale, where EG obtained the worst scores. Moreover, EG women were more sexually distressed than CG women, as revealed in the SDS score.

EG had worse scores in total than CG in the SCL-90-R in the Global Severity Index, Somatization, Obsessive Compulsive, Depression, Anxiety, Phobic Anxiety, and Psychoticism dimensions. Regarding alexithymia (TAS-20), the two groups differed only for the “Difficulties Identifying Feelings” subscale, where EG reached higher scores than CG. EG and CG differed significantly in the “Negative Affects” subscale of PANAS, where EG women scored higher than CG women.

Additionally, EG showed more negative cognitions regarding sexuality than CG. In the SDBQ, EG women presented a higher presence of dysfunctional beliefs in the “Affection Primacy” subscale than CG. A difference was also shown in “Sexual Conservatorism” and in “Age-related Beliefs” subscales, with EG scoring higher than CG (although not significant). In the SMQ, there were no significant differences between groups. Finally, negative schemas were more reported by EG than CG in the “Helpless” domain.

## 4. Discussion

In light of the BPS approach, the aim of the present study was to explore the impact of some cognitive and psycho-emotional factors on sexual functioning, comparing women with and without endometriosis. The two groups were similar in their sociodemographic characteristics with some significant differences. The first distinction concerned the relational status. The majority of women in both groups were in a relationship, while single women were less present in the endometriosis group. This difference might be due to the snowball methodology. Therefore, further studies with a larger number of participants and a better research design are needed. Another difference was the desire to have children, which seemed higher in women with endometriosis. This could be explained by the fact that endometriosis is often associated with infertility problems or difficulty conceiving [2]. Thus, women with endometriosis in our study might be more focused on the desire to become pregnant than women without the pathology [94]. The two groups differed significantly also for the self-declaration of having a sexual problem, where EG women reported suffering from sexual difficulties more than CG ones. This was an expected result since endometriosis is often associated with sexual problems [11,21,23,35,37]. Unexpectedly, just 24.1% of EG women referred to sexual health professionals or were undergoing treatment for these disorders at the time of the study. Nimbi et al. [12], found similar results in a study on GP, suggesting that some factors may act as barriers for seeking help, such as thinking that sex is unimportant or that experiencing GP is normal for women, or that their sexual problems cannot be solved [10,95,96,97,98].

The presence of GPP emerged from the SF-MPQ where 68.96% of women with endometriosis reported having experienced painful symptoms during sexual activities in the last 6 months. In addition, pain intensity was significantly different between the two groups: patients had worse scores than controls. The presence of GPP was not surprising even though women with endometriosis in our study were undergoing a hormonal treatment, which usually helps to mitigate pain. These treatments are useful for relieving pain and improving sexual health only in DIE or in cases where painful symptoms are closely linked to specific sites of lesions [99]. Psycho-social factors could have influenced the worst sexual functioning and the highest GPP in the women who participated in this study, as already reported in previous studies [11,99].

Regarding sexual functioning, no significant differences emerged between the two groups in FSFI scores. In fact, both groups’ mean scores seem to indicate sexual dysfunctions (FSFI total score ≤ 26.55). Sexual dysfunctions are very common in the female general population; a prevalence of 40–50% is estimated [100]. Moreover, the FSFI is strongly influenced by sexual activity that a woman had in the last month and to penetrative sexual intercourse; therefore, the results obtained could not represent the real women’s experience of sexuality. Furthermore, it does not assess sexual distress. In fact, the occurrence of a sexual symptom by it-self is not enough to diagnose a sexual dysfunction; the presence of significant sexual distress is required [10,11]. It follows that FSFI results alone do not provide a real indication about differences in sexual function between patients and controls. Pain was the only FSFI scale in which groups showed significant differences; women with endometriosis having the worst scores. These findings confirm previous research highlighting that dyspareunia is the most common disorder in women with endometriosis [13,21,23,35,36,101].

As pointed out in the literature [41,43], when sexual distress is measured, the prevalence of sexual dysfunctions in patients with endometriosis increases. In line with this, in our study, women with endometriosis showed a higher score on SDS, highlighting that these women were more sexually distressed than control women. This result could also explain the greater percentage of EG women who self-declared to suffer from a sexual problem. Data are in accordance with our first hypothesis and with previous studies [11,21,23,35,37], underling the disease’s influence on patients’ sexual health.

Concerning the presence of psychopathological symptoms, the group of women with endometriosis scored worse than the control group in several domains of the SCL-90-R. These findings confirm that mental health may be severely impaired in women who have this disease [11,28,29]. Anxiety and depression are widely reported in the literature [14,17,19,28,29,30,31,32,33], as well as phobic anxiety, obsessive compulsive and somatization; CPP may lead women to focus more to signals from their body or the situation they are experiencing. This could result in a persistent anxious state, often phobic, especially towards pain (catastrophizing) or obsessive compulsive behaviors to feel control [102]. Somatization was another expected outcome [28,29,33,34]. The Psychoticism domain could denote the presence of a tendency for detachment and retirement, which the pathology could cause. As reported in other studies, women might avoid social situations due to the symptomatology and the feeling of distrust [11,13]. In addition, the burden of the pathology might elicit detachment as a reaction, a splitting mechanism to manage emotions.

Emotional difficulties, such as recognizing feelings (TAS-20), were more present in the endometriosis group than in the control one. However, our hypothesis was only partially confirmed as there were no significant differences in the total score and the other domains of TAS-20. The presence of alexithymia in endometriosis patients has been widely reported in the literature [28,29,30,33], emphasizing that it is a crucial aspect clinicians should take into account when treating these patients. These findings appear to have a link to those obtained in SCL-90-R. On the one hand, difficulties in recognizing feelings may be associated with a tendency to somatization [103,104]; in fact, suppression of emotions seems to correlate with a high level of perceived pain [59]. On the other hand, alexithymic traits could represent a defense mechanism against mental and physical pain (e.g., expression of emotional numbing in post-traumatic stress disorder) [105,106]. Psychopathological traits and alexithymia play a role in female sexual dysfunctions in general [107] and endometriosis-related sexual dysfunctions [28,29,48,108]. Therefore, these data emphasize the importance of assessing them.

The two groups also differed significantly in the presence of negative emotions about sexuality, with higher scores reported by women with endometriosis. These data confirmed studies on GP [12] and other sexual dysfunctions [64,65,68,69,72,73,74,75,109,110,111] in which negative thoughts and feelings (anger, sadness, disappointment, etc.) may distract from the erotic and pleasurable situation, increasing the possibilities of incurring in an unpleasant experience, characterized by a sense of guilt and detachment [69,73,76,109].

Finally, women with and without endometriosis also showed significant differences concerning cognitive factors. Sexual dysfunctional beliefs (SDBQ) were more prevalent in women with endometriosis. These women scored higher only in the “Affection Primacy” domain. These findings were not surprising since women with endometriosis seem to give much importance to the relationship: the presence of a partner has a significant and protective role for women affected by the pathology, acting as an emotional support [24,112]. Although having a partner has a protective role, being focused only on the partner and on the affection can lead women to shift their attention away from their wellbeing and pleasure. Women with these dysfunctional beliefs are more likely to suffer from sexual dysfunctions [67,71,88,89]. Some significant trends are shown in “Sexual Conservatorism” and “Age-related belief”, suggesting the need for more studies to investigate these differences further.

Regarding negative automatic thoughts, usually present in women with sexual difficulties [90,91], this study does not confirm the initial hypothesis; the two groups did not show significant differences. However, women with endometriosis showed greater activation of negative sexual schemas than controls. The “Helpless” sexual schema was significantly more prominent in women affected by the pathology. Previous studies have emphasized the presence of this negative cognitive schema in women with sexual dysfunctions [66,70,92,93]. This finding is in line with previous research [59,60,61,62]: women who feel that they do not have control over their pain and who exhibit a passive coping style usually report greater pain and sexual discomfort. The “Helpless” cognitive schema could be part of the barriers to seeking help mentioned above. Women with endometriosis who experience sexual difficulties may be less likely to ask for help from a health professional because of this feeling of impotence. The delay in diagnosis and the experience of being not believed could lead women to reinforce this sexual schema.

At least to our knowledge, this is one of the very few studies that has explored the role of both cognitive and psycho-emotional factors on the sexual functioning of women with endometriosis, highlighting the need for constant multidisciplinary cooperation between healthcare professionals, psychologists, and organizations in providing assistance and finding possible solutions to the needs of women with endometriosis. The results of this study correlate with treatment options, as the current study strengthens the knowledge of sexual distress and cognitive and psycho-emotional factors experienced by women with endometriosis. In fact, the implications of this study could lead to an expansion of treatments available today that integrate medical therapies and those focused on the patient’s cognitive and psycho-emotional well-being.

The study has limitations that should be mentioned. First of all, all of the measures used were self-report. In addition, the SF-MPQ was adapted by the authors and has not been yet validated. This measure was administrated in conjunction with other validated measures (such as the FSFI) to control the possible lack of validity. Some physical measures to assess GPP are also recommended in future research [113]. Future studies could also focus their attention on the role of the partner, as some relational aspects could have an influence on psychological and emotional state of patients [21,24,25,26,27,112,114]. The presence of infertility, which was not considered in this study, may influence the results [10,54,94]. It would be interesting to investigate whether cognitive and psycho-emotional factors are different in women with and without reproductive problems related to the disease. As regards the control group, the cohort of women reached could be not representative of the female population because of the snowball sampling. This could lead to concerns about the generalization of the results. Moreover, choosing a web-survey could have limited the participation of women less confident and familiar with technologies. Finally, the participants’ answers where anonymous, therefore it could not be possible control if women completed questionnaires many times. However, to limit this bias, all duplicates were removed from database.

## 5. Conclusions

Endometriosis has a great impact on women’s sexual health. However, considering only the presence of symptoms in the sexual response phases could lead to underestimating the real sexual experience in endometriosis patients [11,42,43]. Thus, it is crucial to consider the presence of sexual distress, as women with endometriosis appeared to be more sexually distressed than dysfunctional. Cognitive and psycho-emotional factors seem to be implicated in the low sexual functioning of these patients. Thus, professionals should pay attention to these elements to help women obtain better sexual health. Furthermore, dysfunctional cognitions could lead women not to seek help or not to believe that treatment is possible. Health care practitioners should always assess the presence of GPP and sexual problems because women could avoid speaking spontaneously about it. Sensibilization programs are still needed to increase women’s awareness and correct the wrong beliefs regarding female sexual health.

In the framework of the BPS method, a multidisciplinary approach is strongly recommended [11,58,115,116]. Psychologists, sexologists, pelvic floor physiotherapists, and gynecologists need to collaborate to provide a successful treatment tailored to women’s needs [11,58,115,116].

## Figures and Tables

**Table 1:** Sociodemographic characteristics of the sample.

	CG *n* = 100	EG *n* = 87	
	M ± SD (range)	M ± SD (range)	
Age	34.58 ± 9.27 (18–61)	34.10 ± 9.18 (18–56)	-
	*n* (%)	*n* (%)	
Marital Status			^-^
Unmarried	65 (65.0)	52 (59.8)	
Married	23 (23.0)	28 (32.2)	
Separated	11 (11.0)	7 (8.0)	
Widow	1 (1.0)	0	
Relationship Status			Chi^2^ = 6.031 *
Single	31 (31.0)	14 (16.1)	
In a Relationship (not cohabitant)	24 (24.0)	29 (33.3)	
In a Relationship (cohabitant)	45 (45.0)	44 (50.6)	
Children			-
No	68 (68.0)	64 (73.6)	
Yes	32 (32.0)	23 (26.4)	
Desire of Having a Baby now			Chi^2^ = 5.008 *
No	72 (72.0)	49 (56.3)	
Yes	28 (28.0)	38 (43.7)	
Education Level			-
Primary School	5 (5.0)	6 (6.9)	
Secondary School	37 (37.0)	32 (36.8)	
Degree Of Higher	58 (58.0)	49 (56.3)	
Work Status			-
Unemployed	17 (17.0)	19 (21.8)	
Employed	60 (60.0)	52 (59.8)	
Student	22 (22.0)	16 (18.4)	
Retired	1 (1.0)	0	
Sexual Problems (self-reporting at least a sexual problem in the last 6 months)			Chi^2^ = 20.762 ***
No	81 (81.0)	43 (49.4)	
Yes	19 (19.0)	44 (50.6)	

* *p* < 0.05; *** *p* < 0.001. CG = Control Group. EG = Endometriosis Group.

**Table 2:** Differences between women with and without genital pain: one-way ANOVAs and MANOVAs.

Variable	CG	EG	F_(1, 185)_	*p*	Partial Eta^2^
SF-MPQ total score	22.83 ± 23.89	41.43 ± 19.27	4.879	0.031 *	0.071
FSFI total score (sexual function)	24.81 ± 10.14	22.49 ± 9.66	2.418	0.122	0.013
Desire	3.86 ± 1.19	3.79 ± 1.11	0.152	0.697	0.001
Arousal	3.97 ± 2.01	3.76 ± 1.82	0.524	0.470	0.003
Lubrication	4.52 ± 2.18	4.01 ± 2.18	2.423	0.121	0.014
Orgasm	3.96 ± 2.21	3.62 ± 2.19	1.071	0.302	0.006
Satisfaction	4.09 ± 1.66	4.06 ± 1.66	0.012	0.913	0.000
Pain	4.42 ± 2.33	3.26 ± 2.12	11.890	0.001 ***	0.063
SDS total score (sexual distress)	7.95 ± 9.37	14.49 ± 13.44	14.680	0.000 ***	0.077
SCL-90-R Global Severity Index (psychological problems)	0.69 ± 0.55	0.91 ± 0.65	6.344	0.013 *	0.033
Somatization	0.78 ± 0.69	1.23 ± 0.86	15.818	0.000 ***	0.079
Obsessive Compulsive	0.84 ± 0.66	1.10 ± 0.78	5.617	0.019 *	0.030
Interpersonal sensitivity	0.70 ± 0.66	0.76 ± 0.68	0.413	0.521	0.002
Depression	0.88 ± 0.71	1.19 ± 0.84	7.514	0.007 **	0.039
Anxiety	0.69 ± 0.65	0.90 ± 0.80	4.039	0.046 *	0.021
Hostility	0.60 ± 0.65	0.70 ± 0.67	0.983	0.323	0.005
Phobic anxiety	0.22 ± 0.49	0.39 ± 0.67	4.126	0.044 *	0.022
Paranoid ideation	0.73 ± 0.80	0.81 ± 0.77	0.526	0.469	0.003
Psychoticism	0.39 ± 46	0.54 ± 0.59	3.884	0.050 *	0.021
TAS-20 total score (alexithymia)	41.68 ± 11.42	42.90 ± 12.89	0.468	0.495	0.003
Identifying feelings	13.90 ± 5.77	16.29 ± 6.71	6.845	0.01 **	0.036
Describing feelings	11.81 ± 5.10	11.16 ± 4.36	0.860	0.355	0.005
Externally oriented thoughts	15.97 ± 4.90	15.45 ± 4.72	0.546	0.461	0.003
PANAS (sexuality-related emotions)					
Positive Affects	35.26 ± 8.88	32.89 ± 9.89	2.284	0.133	0.015
Negative Affects	14.72 ± 6.10	18.72 ± 8.97	10.617	0.001 ***	0.066
SDBQ total score (dysfunctional beliefs)	46.21 ± 10.48	51.30 ± 12.77	8.305	0.004 **	0.046
Sexual conservatorism	18.86 ± 4.80	20.66 ± 7.76	3.56	0.061	0.020
Affection Primacy	12.26 ± 3.74	14.18 ± 3.21	12.568	0.001 ***	0.068
Control Over Sexuality	9.50 ± 3.62	10.22 ± 4.05	1.505	0.222	0.009
Age-related Beliefs	5.59 ± 2.14	6.24 ± 2.24	3.817	0.052	0.022
SMQ total score (automatic thoughts)	53.06 ± 14.02	55.21 ± 14.92	0.931	0.336	0.005
Sexual Abuse Thoughts	9.40 ± 2.77	9.33 ± 3.57	0.019	0.891	0.000
Lack Erotic Thoughts	12.77 ± 4.17	13.43 ± 4.49	0.984	0.323	0.006
Low Self-Body Image Thoughts	7.32 ± 3.29	7.31 ± 3.10	0.001	0.977	0.000
Failure Disengagement Thoughts	9.82 ± 3.97	9.35 ± 3.85	1.864	0.174	0.011
Sexual Passivity Control	9.23 ± 3.50	9.76 ± 4.00	0.862	0.354	0.005
Partner Lack Affection	5.33 ± 2.55	5.56 ± 2.54	0.327	0.568	0.002
QCSASC total score (cognitive schemas activation in sexual contexts)	43.15 ± 18.14	47.17 ± 18.02	2.064	0.153	0.012
Helpless	21.72 ± 9.35	25.53 ± 10.82	6.080	0.015 *	0.035
Unlovable	21.43 ± 9.81	21.64 ± 8.55	0.021	0.885	0.000

* *p* < 0.05; ** *p* < 0.01; *** *p* < 0.001. CG = Control Group. EG = Endometriosis Group.

## Data Availability

Data were collected in an encrypted electronic database and were stored at the Institute of Clinical Sexology of Rome, via Savoia, 78 00198 Rome.
